# Detection of relevant pathogens and contaminants in blood cultures after implementation of single-sampling strategy and initial specimen diversion

**DOI:** 10.1007/s10096-025-05196-4

**Published:** 2025-06-25

**Authors:** Karl Oldberg, Fredrik Kahn, Magnus Rasmussen, John Walles

**Affiliations:** 1 Department of Clinical Microbiology, Skåne University Hospital, Lund, Sweden; 2 Division of Infection Medicine, Department of Clinical Sciences, Lund University, Lund, Sweden; 3 Department of Infectious Diseases, Skåne University Hospital, Lund, Sweden; 4 Wallenberg Center for Molecular Medicine, Lund University, Lund, Sweden; 5 Clinical Infection Medicine, Department of Translational Medicine, Lund University, Malmö, Sweden

**Keywords:** Blood culture, Bacteraemia, Contamination, Blood culture sampling

## Abstract

**Purpose:**

Blood culture collection with all bottles filled from a single venipuncture (single-sampling strategy, SSS) is increasingly used. More data on the effects of SSS on blood culture contamination is needed. After combined implementation of SSS and initial specimen diversion (ISDT) in our region, we evaluated the proportion of sampling events with at least four bottles, with growth of relevant pathogens and with growth of coagulase-negative staphylococci (CoNS) before and after the intervention.

**Methods:**

We retrospectively reviewed all blood cultures collected from adults in emergency departments in our region in southern Sweden during six years before and 18 months after the intervention.

**Results:**

Among 117 142 sampling events included in the analysis, the proportion where at least four bottles were obtained increased from 87.6 to 92.3% (*p* < 0.001), while growth of relevant pathogens remained unchanged at 13.6%. In the subset of sampling events with no more than one species, there was a decrease in the proportion with CoNS in 1–2 out of 4 bottles (4.0 to 3.3%, *p* < 0.001), while events with CoNS in 3–4 out of 4 bottles increased (0.39 to 0.49%, *p* = 0.029).

**Conclusion:**

We confirm positive effects of SSS and ISDT on adequate sample volume and a net decrease in the proportion of sampling events with growth of CoNS. Conversely, we observed an increase in CoNS in multiple bottles, probably caused by contamination. This possible drawback with SSS has not been reported before and should be studied further.

**Supplementary Information:**

The online version contains supplementary material available at 10.1007/s10096-025-05196-4.

## Introduction

Blood culture is a key diagnostic tool in severe bacterial infections. The methods for obtaining blood cultures aim at reaching a high sensitivity for detecting bacteria in the blood stream, while keeping the rate of culture bottles contaminated by bacteria from the skin low. A higher sensitivity can be achieved by increasing the sampled blood volume by increasing the number of bottles, and by increasing the volume in each bottle [1–4]. To achieve an adequate sample volume, the typical recommendation the last decades has been to fill an aerobic and an anaerobic bottle with 8–10 mL blood each, from one peripheral venipuncture, and then repeat the procedure with a second venipuncture, with a total of at least four bottles [5]. Recently, this traditional multi-sampling strategy (MSS) has been challenged, and it has been suggested that all bottles can be collected at once, from a single venipuncture (single-sampling strategy, SSS) [6]. Previous studies have indicated a sensitivity using SSS at least as high as with MSS [7–10], a higher proportion of sampling events with at least four bottles [9] and a lower contamination rate [10]. Despite this, it seems plausible that the risk might increase that both blood culture sets are contaminated if both sets are obtained through a single venipuncture, although this remains unexplored. This is of potential importance, as the number of positive bottles is used to assess the risk for true CoNS bacteraemia in some settings [11]. Blood culture contamination rates can be reduced by disinfecting the skin with alcohol-based antiseptic solutions before blood collection, and by training staff [12]. In addition, discarding the first blood portion in specially designed devices or ordinary blood collection tubes (“initial specimen diversion technique” [ISDT]), has been proven effective in reducing the contamination rate [13–15].

Based on these results, the Swedish Society for Clinical Microbiology changed their recommendations in 2022 to a bundle of routines combining SSS and ISDT as the standard method for blood culture collection [16]. The American Clinical and Laboratory Standards Institute still recommends the MSS, and considers ISDT optional [5], while the UK Standards for Microbiology Investigations give conflicting recommendations regarding whether to use SSS or MSS and does not mention ISDT [17]. Our healthcare region implemented combined SSS and ISDT in October 2021.

Here, we evaluate the combined effect of SSS and ISDT on (i) the proportion of sampling events with at least four blood culture bottles, (ii) the proportion of sampling events with growth of relevant pathogens and common contaminants, and (iii) the proportion of sampling events with growth of coagulase-negative staphylococci (CoNS) in 1–2 and 3–4 out of 4 bottles.

## Materials and methods

### Study setting and design

The Department of Clinical Microbiology in Region Skåne serves all 10 hospitals in the region, which has a population of 1.4 million. BD BACTEC FX (Becton Dickinson, Franklin Lakes, New Jersey, USA) is used for blood cultures, with the culture media Lytic Anaerobic, Plus Aerobic and Peds Plus. Blood culture collection was typically performed by antecubital venipuncture, after disinfection with 5 mg/mL chlorhexidine gluconate in 56% ethanol (w/v). Either a “butterfly needle” or a peripheral venous catheter that was just introduced, together with an adapter for vacuum blood collection tubes, was used for drawing blood. Before the intervention, two venipunctures were recommended, with two bottles drawn from each site in the order aerobic-anaerobic. After the intervention, the recommendation was to first fill the ISDT device - a vacuum tube for blood sample collection with disinfected membrane, as described by Patton et al. [12] - that was subsequently discarded, before filling four blood culture bottles in the order aerobic-anaerobic-aerobic-anaerobic. During the whole studied period, incubation of bottles started 24/7 in the five largest hospitals in the region, while the bottles from the smaller hospitals had to be transported to a nearby hospital. Positive bottles were transported to the central microbiology laboratory and their contents were gram stained, streaked onto solid culture media, and species were identified using MALDI-TOF (Bruker Daltonics, Bremen, Germany).

From the database of the Department of Clinical Microbiology, we retrieved anonymous data on all blood cultures obtained from January 2016 to March 2023, from adults (≥ 18 years old) in nine emergency departments (EDs) in Region Skåne that implemented SSS and ISDT October 6th, 2021, and from the single ED in Helsingborg that implemented SSS before 2016. The period was chosen to capture fluctuations over long timespans. The dataset contained patient age, sample date, number and type of blood culture bottles, the organisms growing in the cultures, and requesting clinic. Sampling events with only a single bottle (except paediatric bottles from adults) were excluded due to risk of erroneous registration in the database, and mycosis bottles were excluded as they were deemed irrelevant for the purpose of the study. Data from the Helsingborg ED was only used for comparison with the other EDs regarding proportion of sampling events with at least four bottles.

## Study definitions

A sampling event consisted of all blood culture bottles that were collected from a patient at one date and one clinic. The recommendation for using SSS and ISDT was communicated October 6th, 2021, as a news update on an internal website, a new, digitally available standard operations procedure for blood culture collection, and emails to designated contact persons at all hospital departments. Adopting an intention-to-treat viewpoint, we considered all sampling events before October 6th, 2021, as using MSS, and all sampling events from that date as using SSS and ISDT. “Contaminant species” were defined as any of the following genera or groups of species, regardless of the number of positive bottles: *Bacillus*,* Paenibacillus*,* Lysinibacillus*,* Corynebacterium*,* Cutibacterium*,* Dermabacter*,* Kocuria*,* Kytococcus*,* Micrococcus*, and all CoNS except *S. lugdunensis.* Other findings were considered relevant pathogens. Since CoNS (*S. lugdunensis* excluded) are the most common contaminants, we focused on them in the analyses.

For the analyses of a change in number of bottles per sampling event, and the proportion of sampling events with growth of relevant pathogens, we used the complete dataset. Due to a change of laboratory information system during the study period, stored information regarding blood cultures was more detailed from March 2021, which complicated detailed analyses of the number of positive bottles. To allow for both analysis over a long timespan, and more detailed studies, analysis of CoNS frequency was performed in two separate datasets of four-bottle sampling events:

1. CoNS A spanning January 2016 to March 2023 with negative and monomicrobial sampling events only.
2. CoNS B spanning March 2021 to March 2023 with all sampling events, negative, mono- and polymicrobial, as a sensitivity analysis.

In addition to reporting the proportion of sampling events with 1–2 and 3–4 out of 4 bottles, we made the same univariate analysis for *S. aureus* in the dataset CoNS A for reference.

### Data analysis

Data was presented descriptively, stratified by sampling before vs. after the intervention, using Chi-square test for categorical variables and Mann-Whitney U-test for continuous variables. We performed sensitivity analyses for the outcomes “at least four bottles per sampling event”, “CoNS in 1–2 out of 4 bottles” and “CoNS in 3–4 out of 4 bottles”, with adjustment for patient age and time-variable factors to estimate ED workload, including COVID-19 admissions. These procedures are described in the supplementary material. Statistical analyses were made with R version 4.4.2. The level of statistical significance was *p* < 0.05.

## Ethical considerations

Microbiological data was anonymised with no residual key before it was made available to the researchers, with no possible link to patients. Ethical permission was thus not needed according to Swedish law.

## Results

**Fig. 1:**
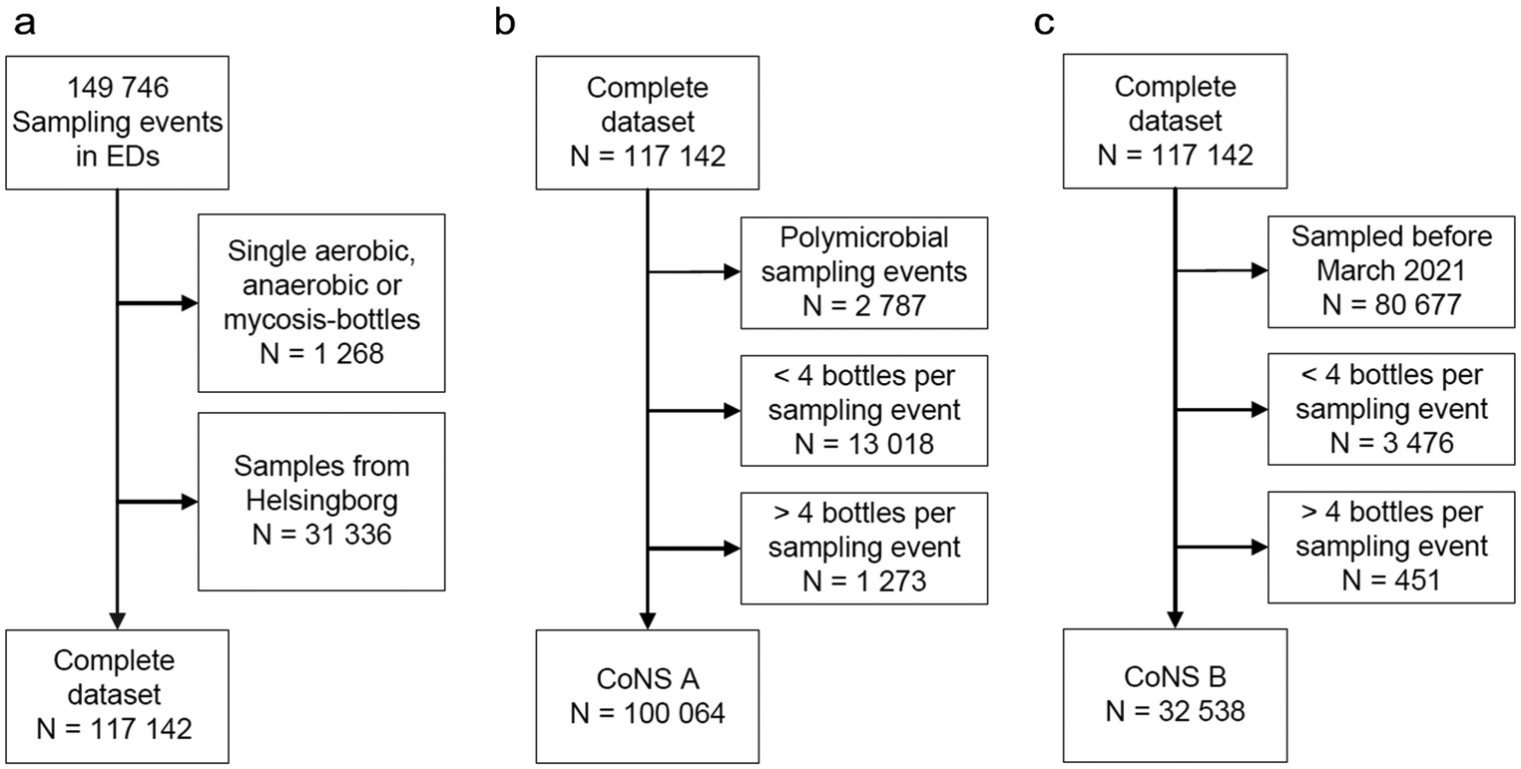
Inclusions and exclusions. Numbers are sampling events. **a**. Exclusions to create the complete dataset. **b**. and **c**. Exclusions to derive the datasets CoNS A and CoNS B from the complete dataset. CoNS A and CoNS B were used for calculations of the proportion of sampling events with growth of coagulase-negative staphylococci in 1–2 or 3–4 out of 4 bottles. EDs = Emergency departments

**Fig. 2:**
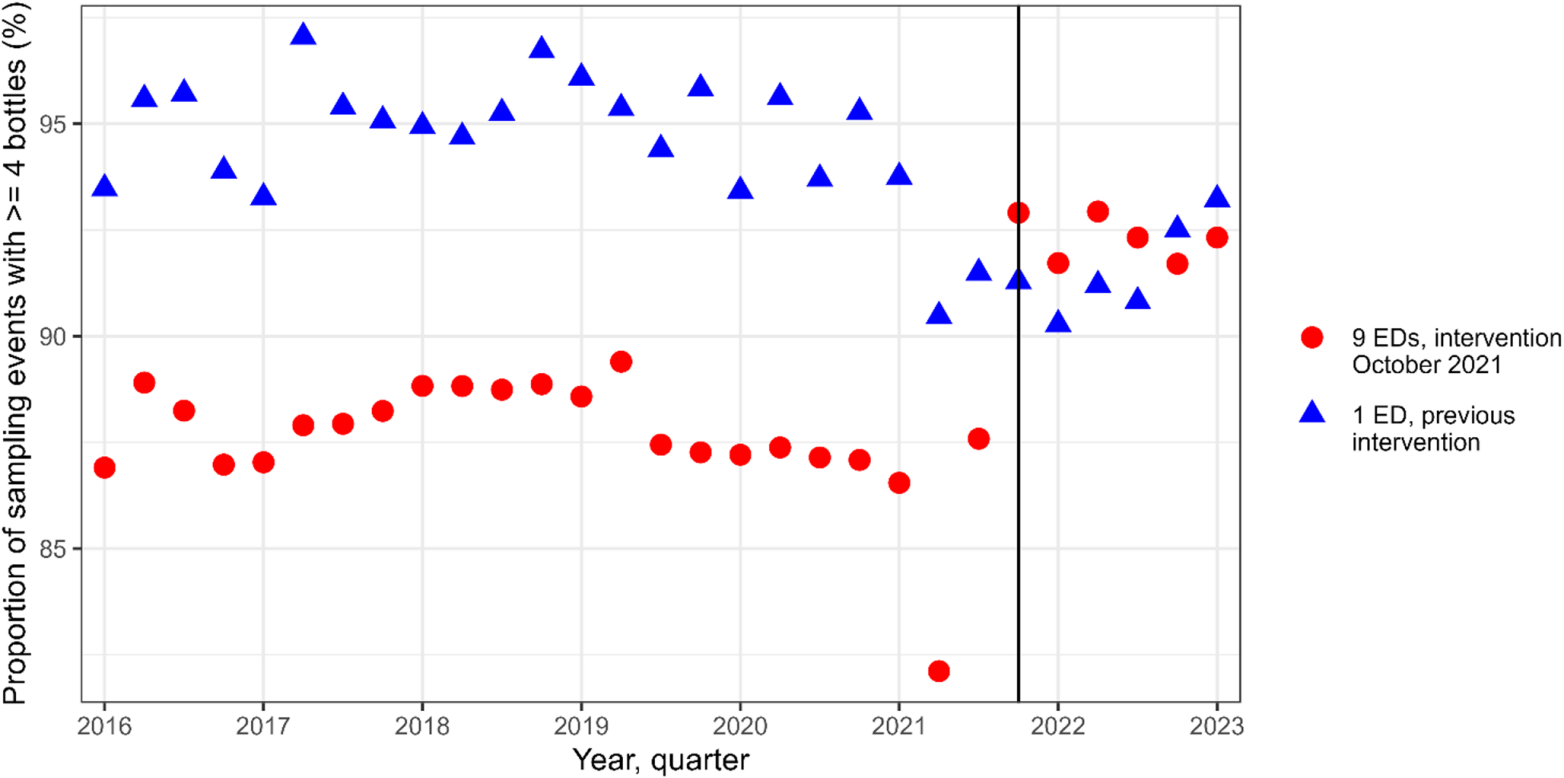
The proportion of sampling events with at least four bottles per quarter. The main dataset is constituted by sampling events in nine emergency departments where the intervention was introduced October 6th, 2021 (black vertical line), while the emergency department in Helsingborg introduced single-sampling strategy before 2016

In total, 149 746 sampling events were reviewed, and 117 142 sampling events remained after exclusions (Fig. 1). There was an increase in the proportion of sampling events with at least four bottles from 87.6 to 92.3% (*p* < 0.001, Table 1), an increase that was not seen in Helsingborg, where SSS was previously implemented (23 602/24 940 [94.6%] before vs. 5 857/6 396 [91.6%] after the intervention, *p* < 0.001) (Fig. 2).

**Table 1:** Age of the patients at sampling, and the proportion of sampling events with relevant pathogens and contaminant species before and after the intervention. These analyses are based on the complete dataset. IQR = interquartile range. CoNS = coagulase-negative Staphylococci

	Before intervention *n* = 89 896	After intervention *n* = 27 246	Univariate *p*
Age, median (IQR)	73 (59–82)	75 (62–83)	*P* < 0.001
At least 4 bottles	78 763 (87.6%)	25 154 (92.3%)	*P* < 0.001
Positive blood culture in sampling event	16 759 (18.6%)	4 989 (18.3%)	*P* = 0.22
Relevant pathogens in sampling event	12 221 (13.6%)	3 697 (13.6%)	*P* = 0.92
Contaminant species in sampling event	5 197 (5.8%)	1 491 (5.5%)	*P* = 0.056
CoNS in sampling event	4 633 (5.2%)	1 318 (4.8%)	*P* = 0.039

There were no significant differences in the proportion of sampling events with positive bottles or with growth of relevant pathogens after SSS implementation (Table 1). The proportion of sampling events with contaminant species was 5.8% before and 5.5% after the intervention (*p* = 0.056), the decrease largely attributed to reduced retrieval of CoNS (5.2–4.8%, *p* = 0.039, Table 1).

**Fig. 3:**
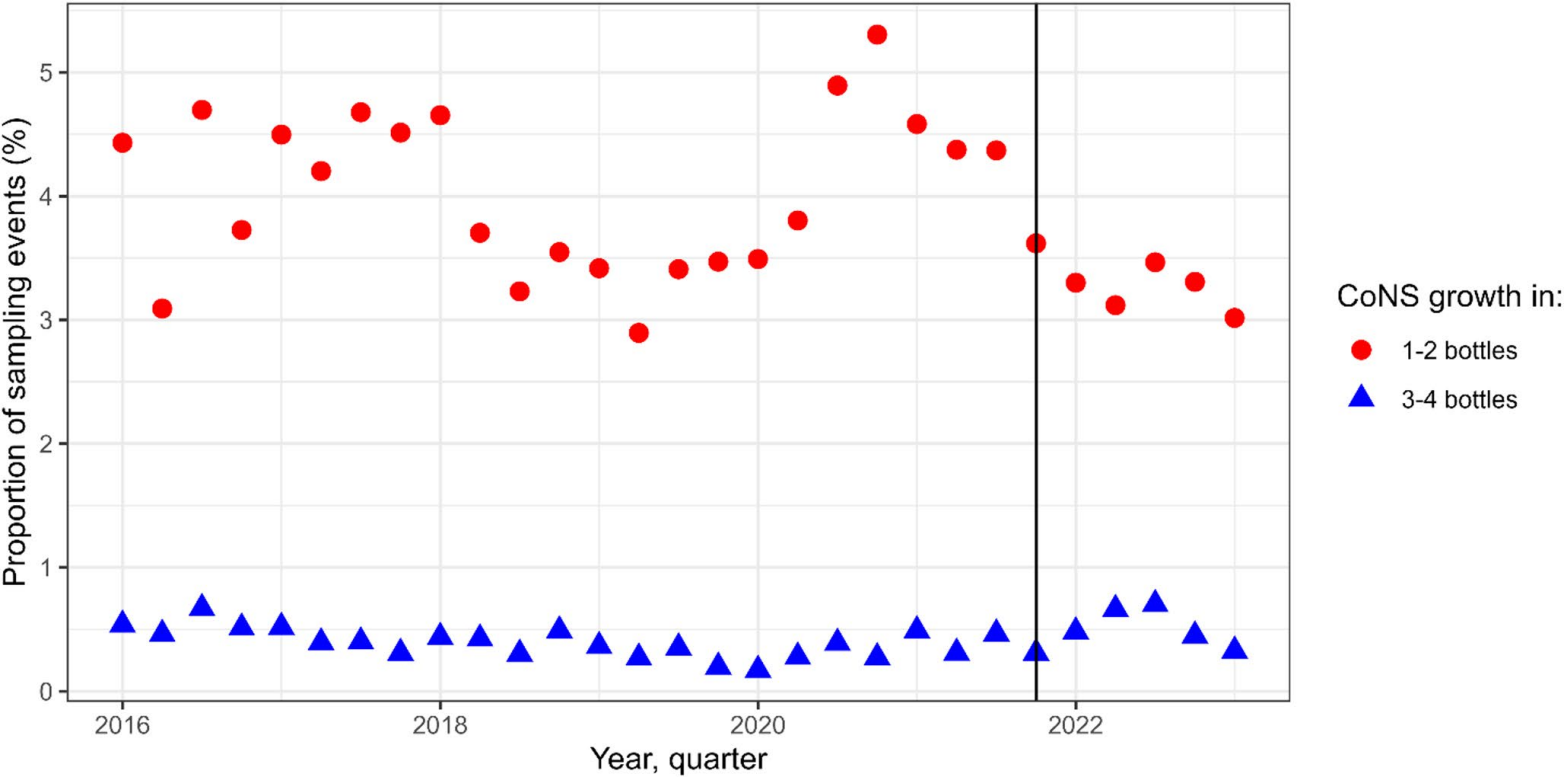
The proportions of sampling events with growth of coagulase-negative staphylococci (CoNS) in 1–2 and 3–4 out of 4 bottles, per quarter. The analysis is based on the dataset CoNS A, with negative and monomicrobial infections. The black vertical line marks the quarter when the intervention was made

In dataset CoNS A, the proportion of sampling events with growth of CoNS in 1–2 out of 4 bottles decreased from 4.0 to 3.3% (*p* < 0.001), while the proportion with CoNS in 3–4 out of 4 bottles increased from 0.39 to 0.49% (*p* = 0.029, Table 2). As a reference to these results, we analysed the proportion of sampling events with growth of *S. aureus* in 1–2 bottles (0.43% before and 0.46% after, *p* = 0.61), and 3–4 out of 4 bottles (1.1% before and 1.1% after, *p* = 0.76). The increase in CoNS in 3–4 out of 4 bottles corresponds to an increase by 24 sampling events with monomicrobial growth of CoNS in three or four bottles, during 18 months after the intervention. The progression of the two CoNS-proportions during the studied period is displayed graphically in Fig. 3. In dataset CoNS B, the proportion of sampling events with CoNS in 1–2 out of 4 bottles decreased from 5.2 to 3.8% (*p* < 0.001), while the proportion with CoNS in 3–4 out of 4 bottles increased from 0.62 to 1.0% (*p* = 0.0016, Supplementary Table S1). This corresponds to 91 additional mono- or polymicrobial sampling events with CoNS in 3–4 out of 4 bottles during 18 months after SSS implementation. The sensitivity analyses, with adjustment for patient age and time-variable factors to estimate ED workload, did not change any of the results (Supplementary tables S2– S9).

**Table 2:** The proportion of sampling events with coagulase-negative Staphylococci (CoNS) and the reference relevant pathogen *Staphylococcus aureus* before and after the intervention. The analysis is based on the dataset CoNS A, including negative and monomicrobial sampling events 2016 to 2023. IQR = interquartile range

	Before intervention *n* = 75 872	After intervention *n* = 24 192	Univariate *p*
Age, median (IQR)	73 (59–82)	75 (61–82)	*P* < 0.001
CoNS in sampling event	3 353 (4.4%)	920 (3.8%)	*P* < 0.001
CoNS in 1–2 bottles	3 060 (4.0%)	801 (3.3%)	*P* < 0.001
CoNS in 3–4 bottles	293 (0.39%)	119 (0.49%)	*P* = 0.029
Reference pathogen			
*S. aureus* in sampling event	1 129 (1.5%)	373 (1.5%)	*P* = 0.55
*S. aureus* in 1–2 bottles	330 (0.43%)	112 (0.46%)	*P* = 0.61
*S. aureus* in 3–4 bottles	799 (1.1%)	261 (1.1%)	*P* = 0.76

## Discussion

Following implementation of SSS and ISDT at nine EDs in southern Sweden, we observed an increased proportion of sampling events that included at least four bottles. Overall, we observed a lower proportion of sampling events with growth of CoNS, which was caused by a reduction of sampling events with growth of CoNS in 1–2 out of 4 bottles, but we also observed an increase in the proportion of sampling events with growth of CoNS in 3–4 out of 4 bottles. This increase has not been reported previously and merits further exploration.

Previous studies have observed increased or similar detection of relevant pathogens following SSS implementation, supporting a non-inferior blood culture sensitivity [7–10]. Ekwall-Larsson et al. found an increased sensitivity due to an increase in the proportion of sampling events with at least four bottles from 71 to 96% [9]. In the present study, we observed an increase by 4.7% units in the proportion of sampling events with at least four bottles, from which it is reasonable to expect increased detection of relevant pathogens. However, no significant increase in detection was observed, probably because the increase by 4.7% units from 87.6% is relatively small. The percentage of sampling events with at least four bottles varies substantially between countries and hospitals, with examples from published data of > 85% in a hospital in the United States [18], 71% in a Swedish emergency department [9] and 44% in an Australian emergency department [19]. Hospitals with lower percentages than ours could likely expect a greater increase in the recovery of relevant pathogens after introducing SSS, while those with higher percentages are less likely to see a significant increase. A plausible explanation for the increase in sampling events with at least four bottles, is that it is easier for personnel to draw the blood sample from a single venipuncture when using SSS. Ekwall-Larsson et al. also demonstrated a higher blood volume per bottle after introducing SSS, which should contribute to increased sensitivity [9]. Unfortunately, we lack data on blood volume.

It has been suggested that SSS could lead to fewer blood culture contaminations compared to MSS, as each venipuncture is a risk of introducing skin bacteria into the bottles [20]. However, only one study has demonstrated a reduction in contamination rate using SSS [10], while several studies demonstrate that using an ISDT can reduce the proportion of contaminated blood cultures [13–15]. In the present study, we used the number of positive bottles with CoNS as a marker for contamination: When monitoring contamination rate, a commonly used proxy for a contaminated blood culture is growth of common skin commensals in only one out of at least two blood culture sets, each set containing one aerobic and one anaerobic bottle [5]. SSS makes this definition obsolete, as all collected bottles can be regarded as a one set. Counting the number of positive bottles instead has been described as a method to discern contaminations [11], and the Swedish Society for Clinical Microbiology recommends growth of CoNS in one or two out of four bottles to be used as a metric for monitoring contamination rate [16]. In this study, using a combination of SSS and ISDT, we observed a significant reduction in the crude proportion of sampling events with growth of CoNS. This reduction was attributable to decreased proportions of sampling events with growth of CoNS in 1–2 out of 4 bottles in both studied datasets, in crude analyses and in multivariable models. However, we also observed an increase in the proportion of sampling events with CoNS in 3–4 out of 4 bottles after the intervention, an effect that has not been described before in studies on SSS or ISDT. There were no changes in the proportion of sampling events with *S. aureus* in 1–2 or 3–4 out of 4 bottles, which would have been expected if the changed sampling protocol per se led to different growth dynamics in true bacteraemia, with a change in the number of positive bottles. The remaining possibilities to explain our observations are a change in contamination rate or a change in the prevalence of true CoNS-bacteraemia. Studies on how to interpret the number of bottles with growth of CoNS in a clinical setting have reached conflicting results, with Leyssene et al. [11] finding a correlation between a higher number of positive bottles and true bacteraemia, while Mirrett et al. [21] found no such correlation. The present study was not designed to study the correlation between true CoNS-bacteraemia and the number of positive bottles but adopting the hypothesis that many positive bottles are associated with true bacteraemia, one would expect the prevalence of true CoNS-bacteraemia to have increased after the intervention. We consider it improbable that the prevalence of true infection caused by CoNS, among patients presenting at EDs, increased significantly during the studied period: The increase in sampling events with CoNS in 3–4 out of 4 bottles increased by 26% using the dataset CoNS A, and even more using CoNS B. We are not aware of any changes in the routines of our healthcare system, that would have resulted in a change of that magnitude in the proportion of patients with true CoNS-bacteraemia at EDs. Furthermore, in similarity with CoNS, *S. aureus* is also a common aetiology in infections in intravascular foreign material and permanent venous catheters. If the proportion of patients presenting at EDs with such infections had truly increased significantly, some increase in the proportion with *S. aureus* in blood cultures would also be expected. This proportion remained unchanged, however. Consequently, we find it probable that the changes in CoNS detection we observed, are mainly driven by a decrease in contamination events that affect 1–2 bottles, but an increase in contaminations affecting 3–4 out of 4 bottles. It is probable that previous studies on SSS have had insufficient statistical power to detect this change, since our study by far has the largest sample size. A possible link between SSS and an increased rate of contamination in more than two bottles is, that if a venipuncture is performed with inadequate technique, the risk of contaminating all four bottles is higher than if a second venipuncture is used for the last two bottles. It is difficult to propose a mechanism for how ISDT by itself could increase the risk for contamination in multiple bottles, and we find it unlikely that it contributes to our observation.

Despite the limited evidence for a correlation between multiple positive bottles and true bacteraemia, our experience is that in our region the number of positive bottles is often used to estimate the probability of true CoNS-bacteraemia. Therefore, an increase in contaminating CoNS in multiple bottles, interpreted as significant bacteraemia, can have serious negative effects for patients. The true cause of disease may remain unrecognised and untreated, while unnecessary antibacterial treatment directed towards CoNS and increased time in hospital, is both costly and imposes a risk for in-hospital injuries [22]. Our results motivate that findings of CoNS in multiple bottles are interpreted with due caution.

The change in sampling recommendations in our region was not accompanied by a dedicated practical training campaign, and it is possible that the type of contamination we believe that we can observe, could have been prevented by adequately training personnel, including the proper use of ISDT. The increased proportion of CoNS in 3–4 out of 4 bottles, which we observe after the intervention, corresponded to an absolute number of 24 monomicrobial sampling events during 18 months. It can be questioned whether such an increase is of relevance, but we believe that total effect on the level of regions, health care systems or countries could be a concern. However, the possible increase in multiple contaminated bottles must also be weighed against the positive aspects, namely an increase in the proportion of sampling events with adequate number of bottles, reduced time consumption collecting the samples, reduced patient discomfort and a possible net decrease in contamination rate. Further studies are motivated to investigate the possible effects of SSS on the risk of CoNS contamination in 3–4 bottles, and how to interpret the number of positive bottles.

Strengths in this study is the sample size, which is significantly larger than that of the similar study by Ekwall-Larsson et al. [9]. The present study also covers a longer time span, which has the benefit that fluctuations over time can be observed. Our study is the first that we are aware of to evaluate the combined intervention of SSS and ISDT, a combination that is compelling as it might be an easy way to both decrease time consumption when collecting blood cultures, and to reduce the contamination rate. Finally, it is the first study to evaluate an introduction of SSS and ISDT in multiple hospitals. Such an introduction might be associated with more difficulties regarding communication and education of all staff, than in a setting where only one hospital or department changes its sampling routines. The possible increase in contaminations affecting multiple bottles that we observed might be an example of that. Our results may therefore be more relevant when implementation in whole regions, healthcare systems or countries is considered.

A fundamental limitation in this study is the retrospective design, which makes it difficult to infer causality between the intervention and the observations. We strived to make the conclusions more solid by using two datasets that differed in timeframe and whether sampling events with multiple species were included or not. Secondly, in sensitivity analyses that are presented in the supplementary materials, we adjusted for possible time-variable confounders reflecting ED strain and regional COVID-19 admissions, since the peak of the pandemic occurred within the studied timeframe. Another major limitation is that since SSS and ISDT were introduced simultaneously, it was not possible to separate the effects of each intervention. This study should be regarded as an evaluation of both methods in combination. Furthermore, we lacked information on the sampling method for individual blood cultures. Applying an intention-to-treat perspective, we chose to consider all cultures with sample date before the intervention as collected with the MSS and all cultures afterwards as collected with the SSS and ISDT. It is probable that MSS continued to be used during several days or weeks after the day when the new sampling routines were communicated, but in a decreasing frequency. However, the change in the proportion of sampling events with at least four bottles that can be seen in Fig. 2 at the time of the intervention, suggests a rapid endorsement of the new routines, since sampling with at least four bottles was recommended also before the intervention. We also lacked information on whether sampling was made through central venous catheters instead of peripheral venipuncture. We tried to reduce the number of such sampling events by only including samples from EDs, but a small proportion of the sampling events both before and after the intervention are likely to include bottles filled from different types of permanent venous catheters. We do not believe that there has been a systematic change in the frequency of sampling from catheters during the studied period. If anything, the recommendations for always using peripheral venipuncture whenever possible, were clarified at the time of the intervention. Theoretically, this could have led to a slightly decreased proportion of sampling events with CoNS, as sampling through catheters imposes a higher risk for contamination. Finally, due to the study design with anonymous data, we were not able to make individual assessments on whether growth of CoNS represented true bacteraemia or contamination. Such an assessment could have given more reliable information on whether the increase of CoNS in multiple bottles was caused by an increase in the rate of true CoNS infections or an increase in contaminations.

In conclusion, the combined introduction of SSS and ISDT in blood culture collection resulted in a higher proportion of sampling events with at least four bottles. CoNS-growth in 1–2 out of 4 bottles decreased but there was an increase in sampling events with growth of CoNS in 3–4 out of 4 bottles, probably as a result of a changed behaviour of contaminations. This deserves further study and highlights the importance of adequate training of staff collecting blood cultures.

## Electronic supplementary material

Below is the link to the electronic supplementary material.

Supplementary Material 1

## Data Availability

The datasets analysed during the current study are not publicly available as a precaution due to the origin in sensitive data. On reasonable request, the dataset is available from the corresponding author.
