# Supplementary material for: Detection of relevant pathogens and contaminants in blood cultures after implementation of single-sampling strategy and initial specimen diversion
Source: Eur J Clin Microbiol Infect Dis. 2025 Jun 25;44(9):2275–82. doi: 10.1007/s10096-025-05196-4 (PMC12457491; doi:10.1007/s10096-025-05196-4)
Supplement: Supplementary file 1 — Supplementary Material 1 [file 10096_2025_5196_MOESM1_ESM.pdf]

Detection of relevant pathogens and contaminants in blood cultures after implementation of single-sampling strategy and initial specimen diversion

Supplementary material

Authors

Karl Oldberg[1, 2], Fredrik Kahn[2, 3, 4], Magnus Rasmussen[2, 3] and John Walles[1, 5]

Affiliations

1. Department of Clinical Microbiology, Skåne University Hospital, Lund, Sweden
2. Division of Infection Medicine, Department of Clinical Sciences, Lund University, Lund, Sweden
3. Department of Infectious Diseases, Skåne University Hospital, Lund, Sweden
4. Wallenberg Center for Molecular Medicine, Lund University, Lund, Sweden
5. Clinical Infection Medicine, Department of Translational Medicine, Lund University, Malmö, Sweden

Supplementary methods

To complement the analysis of coagulase-negative staphylococci (CoNS) in 1-2 and 3-4 out of 4 bottles with the more traditional monitoring metric for contamination, we analysed the crude proportion of sampling events with growth of CoNS in 1 or 2 out of 2 blood culture sets, for the whole study period, without exclusion of polymicrobial events.

Sensitivity analyses

We hypothesised that the proportion of sampling events with at least four bottles, and the proportion with CoNS could be affected by patient age, emergency department (ED) strain, and the COVID-19 pandemic, all of which could change systematically within the studied timeframe. To adjust the estimation of the intervention effect for these variables, data on monthly median waiting time among patients with suspected infectious diseases in each ED (in quartiles), and weekly total COVID-19 admissions in our region (categorically, with breaks at percentiles

5, 25, 50 and 75), were obtained from open sources at the Swedish National Board of Health and Welfare [1]. Binary logistic regression models were made for the outcomes “at least four bottles per sampling event”, “CoNS in 1-2 out of 4 bottles” and “CoNS in 3-4 out of 4 bottles”, with sample date after the intervention as the exposure variable. Adjusted models included age (categorical, in quartiles), monthly ED waiting time and, for the dataset CoNS B, weekly COVID-19 admissions. Observations missing data on ED waiting time were excluded from the models.

Statistical analyses were made with R version 4.4.2.

Supplementary results

The proportion of sampling events with growth of CoNS in 1 or 2 out of 2 blood culture sets was studied, i.e the traditional method of monitoring contamination rate. Before the intervention, 3 376 / 77 748 (4.3%) sampling events grew CoNS in one set and 1 209 / 24 480 (3.3%) sampling events after the intervention ($p < 0.001$). For CoNS in 2 out of 2 blood culture sets, the numbers were 752 / 77 748 (1.0%) sampling events before and 401 / 24 480 (1.6%) sampling events after the intervention ($p < 0.001$).

The results of the sensitivity analyses are presented in Supplementary tables S1 to S9.

Supplementary tables

	Before intervention<br>n = 7 698	After intervention<br>n = 24 840	Univariate p
Age, median (IQR)	74 (60 – 82)	75 (62 – 83)	P = < 0.001
CoNS in sampling event (%)	452 (5.9 %)	1 209 (4.9 %)	P < 0.001
CoNS in 1-2 bottles	404 (5.2 %)	954 (3.8 %)	P < 0.001
CoNS in 3-4 bottles	48 (0.62%)	255 (1.0%)	P = 0.0016

Supplementary Table S1. The proportion of sampling events with coagulase-negative staphylococci (CoNS) before and after the intervention. The analysis is based on the dataset CoNS B. IQR = interquartile range.

Variable	< 4 bottles n = 12 110	≥ 4 bottles n = 91 674	Odds ratio (95% CI)	p	Adjusted odds ratio (95% CI)	Multivariable model p
Pre-intervention	10 302 (85%)	69 502 (76%)			Ref	
Post-intervention	1 808 (15%)	22 172 (24%)	1.82 (1.73 – 1.92)	P < 0.001	1.81 (1.72 – 1.92)	P < 0.001
ED waiting, quartile 1	2 841 (24%)	19 194 (21%)			Ref	
ED waiting, quartile 2	3 221 (27%)	23 503 (26%)			1.05 (0.996 – 1.11)	P = 0.071
ED waiting, quartile 3	3 175 (26%)	24 815 (27%)			1.03 (0.974 – 1.09)	P = 0.30
ED waiting, quartile 4	2 873 (24%)	24 162 (26%)			1.02 (0.961 – 1.08)	P = 0.55
Age quartile 1	3 346 (28%)	23 437 (26%)			Ref	
Age quartile 2	3 094 (26%)	23 575 (26%)			1.08 (1.03 – 1.14)	P = 0.0028
Age quartile 3	2 811 (23%)	23 195 (25%)			1.15 (1.09 – 1.22)	P < 0.001
Age quartile 4	2 859 (24%)	21 467 (23%)			1.05 (1.00 – 1.11)	P = 0.051

Supplementary Table S2. Binary logistic regression with at least four bottles per sampling event as the outcome. The analysis is based on the whole dataset, 11 930 observations missing data in emergency department waiting time were excluded. The model includes the variables emergency department waiting time and age. ED = emergency department. 95% CI = 95% confidence interval.

Variable	< 4 bottles n = 3 092	≥ 4 bottles n = 29 173	Odds ratio (95% CI)	P	Adjusted odds ratio (95% CI)	Multivariable model p
Pre-intervention	1 274 (41%)	7 001 (24%)			Ref	
Post-intervention	1 808 (59%)	22 172 (76%)	2.23 (2.07 – 2.41)	P < 0.001	2.46 (2.24 – 2.69)	P < 0.001
ED waiting, quartile 1	415 (14%)	2 643 (9.1%)			Ref	
ED waiting, quartile 2	375 (12%)	3 651 (13%)			1.01 (0.854 – 1.19)	P = 0.93
ED waiting, quartile 3	873 (28%)	8 910 (31%)			1.02 (0.887 – 1.17)	P = 0.78
ED waiting, quartile 4	1 419 (46%)	13 969 (48%)			0.992 (0.867 – 1.13)	P = 0.91
Age quartile 1	830 (27%)	7 380 (25%)			Ref	
Age quartile 2	773 (25%)	7 704 (26%)			1.11 (0.998 – 1.23)	P = 0.056
Age quartile 3	705 (23%)	7 142 (25%)			1.11 (1.00 – 1.24)	P = 0.049
Age quartile 4	774 (25%)	6 947 (24%)			0.972 (0.876 – 1.08)	P = 0.59
COVID-19 hospital occupancy score 1	95 (3.1%)	757 (2.6%)			Ref	
COVID-19 hospital occupancy score 2	329 (11%)	2618 (9.0%)			0.792 (0.618 – 1.01)	P = 0.062
COVID-19 hospital occupancy score 3	927 (30%)	9 388 (32%)			0.660 (0.519 – 0.831)	P < 0.001
COVID-19 hospital occupancy score 4	978 (32%)	9 174 (32%)			0.598 (0.469 – 0.756)	P < 0.001
COVID-19 hospital occupancy score 5	753 (24%)	7 236 (25%)			0.586 (0.458 – 0.743)	P < 0.001

Supplementary Table S3. Binary logistic regression with at least four bottles per sampling event as the outcome. Whole dataset, truncated before March 2021. 4 210 observations missing data in ED waiting time were excluded. The model includes the variables emergency department waiting time, age and COVID-19 occupancy score. ED = emergency department. 95% CI = 95% confidence interval.

Variable	No CoNS n = 84 316	CoNS in 1-2 /4 bottles n = 3 467	Odds ratio (95% CI)	p	Adjusted odds ratio (95% CI)	Multivariable model p
Pre-intervention	63 809 (76%)	2 781 (80%)			Ref	
Post-intervention	20 507 (24%)	686 (20%)	0.768 (0.704 – 0.835)	P < 0.001	0.757 (0.691 – 0.828)	P < 0.001
ED waiting, quartile 1	17 661 (21%)	770 (22%)			Ref	
ED waiting, quartile 2	21 603 (26%)	881 (25%)			0.948 (0.859 – 1.05)	P = 0.29
ED waiting, quartile 3	22 777 (27%)	970 (28%)			1.03 (0.938 – 1.14)	P = 0.50
ED waiting, quartile 4	22 275 (26%)	846 (24%)			0.963 (0.867 – 1.07)	P = 0.48
Age quartile 1	22 054 (26%)	627 (18%)			Ref	
Age quartile 2	21 528 (26%)	916 (26%)			1.50 (1.35 – 1.66)	P < 0.001
Age quartile 3	21 187 (25%)	938 (27%)			1.57 (1.42 – 1.74)	P < 0.001
Age quartile 4	19 547 (23%)	986 (28%)			1.79 (1.61 – 1.98)	P < 0.001

Supplementary Table S4. Binary logistic regression with coagulase-negative staphylococci (CoNS) in 1-2 out of 4 bottles as the outcome. The analysis is based on the dataset CoNS A, 11 930 observations missing data in ED waiting time were excluded, as well as 352 observations with CoNS in 3-4 out of 4 bottles. The model includes the variables emergency department waiting time and age. ED = emergency department. 95% CI = 95% confidence interval.

Variable	No CoNS n = 27 291	CoNS in 1-2 /4 bottles n = 1 191	Odds ratio (95% CI)	p	Adjusted odds ratio (95% CI)	Multivariable model p
Pre-intervention	6 460 (24%)	365 (31%)			Ref	
Post-intervention	20 831 (76%)	826 (69%)	0.702 (0.619 – 0.797)	P < 0.001	0.690 (0.605 – 0.787)	P < 0.001
ED waiting, quartile 1	2 476 (9.1%)	119 (10%)			Ref	
ED waiting, quartile 2	3 432 (13%)	129 (11%)			0.877 (0.677 – 1.14)	P = 0.32
ED waiting, quartile 3	8 317 (31%)	354 (30%)			1.02 (0.821 – 1.28)	P = 0.86
ED waiting, quartile 4	13 066 (48%)	589 (50%)			1.06 (0.865 – 1.31)	P = 0.57
Age quartile 1	7 321 (27%)	242 (20%)			Ref	
Age quartile 2	6 842 (25%)	295 (25%)			1.31 (1.10 – 1.56)	P = 0.0023
Age quartile 3	6 642 (24%)	329 (28%)			1.51 (1.27 – 1.79)	P < 0.001
Age quartile 4	6 486 (24%)	325 (27%)			1.53 (1.30 – 1.82)	P < 0.001

Supplementary Table S5. Binary logistic regression with coagulase-negative staphylococci (CoNS) in 1-2 out of 4 bottles as the outcome. The analysis is based on the dataset CoNS B, 3 785 observations missing data in ED waiting time were excluded, as well as 269 observations with CoNS in 3-4 out of 4 bottles. The model includes the variables emergency department waiting time and age. ED = emergency department. 95% CI = 95% confidence interval.

Variable	No CoNS n = 27 291	CoNS in 1-2 / 4 bottles n = 1 191	Odds ratio (95% CI)	p	Adjusted odds ratio (95% CI)	Multivariable model p
Pre-intervention	6 460 (24%)	365 (31%)			Ref	
Post-intervention	20 831 (76%)	826 (69%)	0.702 (0.619 – 0.797)	P < 0.001	0.705 (0.606 – 0.823)	P < 0.001
ED waiting, quartile 1	2 476 (9.1%)	119 (10%)			Ref	
ED waiting, quartile 2	3 432 (13%)	129 (11%)			0.900 (0.685 – 1.18)	P = 0.45
ED waiting, quartile 3	8 317 (31%)	354 (30%)			1.03 (0.818 – 1.29)	P = 0.83
ED waiting, quartile 4	13 066 (48%)	589 (50%)			1.06 (0.853 – 1.33)	P = 0.60
Age quartile 1	7 321 (27%)	242 (20%)			Ref	
Age quartile 2	6 842 (25%)	295 (25%)			1.31 (1.10 – 1.56)	P = 0.0023
Age quartile 3	6 642 (24%)	329 (28%)			1.51 (1.27 – 1.79)	P < 0.001
Age quartile 4	6 486 (24%)	325 (27%)			1.54 (1.30– 1.82)	P < 0.001
COVID-19 hospital occupancy score 1	687 (2.5%)	48 (4.0%)			Ref	
COVID-19 hospital occupancy score 2	2 435 (8.9%)	121 (10%)			0.806 (0.572 – 1.15)	P = 0.23
COVID-19 hospital occupancy score 3	8 819 (32%)	353 (30%)			0.772 (0.556 – 1.09)	P = 0.13
COVID-19 hospital occupancy score 4	8 550 (31%)	388 (33%)			0.869 (0.623 – 1.23)	P = 0.42
COVID-19 hospital occupancy score 5	6 800 (25%)	281 (24%)			0.794 (0.565 – 1.14)	P = 0.20

Supplementary Table S6. Binary logistic regression with coagulase-negative staphylococci (CoNS) in 1-2 out of 4 bottles as the outcome. The analysis is based on the dataset CoNS B, 3 785 observations missing data in ED waiting time were excluded, as well as 269 observations with CoNS in 3-4 out of 4 bottles. The model includes the variables emergency department waiting time, age and COVID-19 occupancy score. ED = emergency department. 95% CI = 95% confidence interval.

Variable	No CoNS n = 84 316	CoNS in 3-4 / 4 bottles n = 351	Odds ratio	P	Adjusted odds ratio (95% CI)	Multivariable model p
Pre-intervention	63 809 (76%)	256 (73%)			Ref	
Post-intervention	20 507 (24%)	95 (27%)	1.15 (0.908 – 1.46)	P = 0.23	1.15 (0.886 – 1.47)	P= 0.29
ED waiting, quartile 1	17 661 (21%)	64 (18%)			Ref	
ED waiting, quartile 2	21 603 (26%)	98 (28%)			1.24 (0.905 – 1.71)	P = 0.18
ED waiting, quartile 3	22 777 (27%)	95 (27%)			1.11 (0.801 – 1.54)	P = 0.54
ED waiting, quartile 4	22 275 (26%)	94 (27%)			1.10 (0.784 – 1.54)	P = 0.60
Age quartile 1	22 054 (26%)	51 (15%)			Ref	
Age quartile 2	21 528 (26%)	113 (32%)			2.27 (1.64 – 3.18)	P < 0.001
Age quartile 3	21 187 (25%)	83 (24%)			1.69 (1.19 – 2.41)	P = 0.0034
Age quartile 4	19 547 (23%)	104 (30%)			2.29 (1.65 – 3.23)	P < 0.001

Supplementary Table S7. Binary logistic regression with coagulase-negative staphylococci (CoNS) in 3-4 out of 4 bottles as the outcome. The analysis is based on the dataset CoNS A, 11 930 observations missing data in ED waiting time were excluded, as well as 3 467 observations with CoNS in 1-2 out of 4 bottles. The model includes the variables emergency department waiting time and age. ED = emergency department. 95% CI = 95% confidence interval.

Variable	No CoNS n = 27 291	CoNS in 3-4 / 4 bottles n = 269	Odds ratio (95% CI)	p	Adjusted odds ratio (95% CI)	Multivariable model p
Pre-intervention	6 460 (24%)	45 (17%)			Ref	
Post-intervention	20 831 (76%)	224 (83%)	1.54 (1.13 – 2.15)	P = 0.0081	1.41 (1.03 – 1.98)	P = 0.041
ED waiting, quartile 1	2 476 (9.1%)	14 (5.2%)			Ref	
ED waiting, quartile 2	3 432 (13%)	37 (14%)			1.66 (0.909 – 3.21)	P = 0.11
ED waiting, quartile 3	8 317 (31%)	96 (36%)			1.73 (1.00 – 3.21)	P = 0.063
ED waiting, quartile 4	13 066 48%)	122 (45%)			1.42 (0.835 – 2.61)	P = 0.23
Age quartile 1	7 321 (27%)	44 (16%)			Ref	
Age quartile 2	6 842 (25%)	62 (23%)			1.50 (1.02 – 2.23)	P = 0.039
Age quartile 3	6 642 (24%)	76 (28%)			1.88 (1.30 – 2.75)	P < 0.001
Age quartile 4	6 486 (24%)	87 (32%)			2.19 (1.53 – 3.18)	P < 0.001

Supplementary Table S8. Binary logistic regression with coagulase-negative staphylococci (CoNS) in 3-4 out of 4 bottles as the outcome. The analysis is based on the dataset CoNS B, 3 785 observations missing data in ED waiting time were excluded, as well as 1 191 observations with CoNS in 1-2 out of 4 bottles. The model includes the variables emergency department waiting time and age. ED = emergency department. 95% CI = 95% confidence interval.

Variable	No CoNS n = 27 291	CoNS in 3-4 / 4 bottles n = 269	Odds ratio (95% CI)	P	Adjusted odds ratio (95% CI)	Multivariable model p
Pre-intervention	6 460 (24%)	45 (17%)			Ref	
Post-intervention	20 831 (76%)	224 (83%)	1.54 (1.13 – 2.15)	P = 0.0081	1.52 (1.04 – 2.27)	P = 0.037
ED waiting, quartile 1	2 476 (9.1%)	14 (5.2%)			Ref	
ED waiting, quartile 2	3 432 (13%)	37 (14%)			1.62 (0.873 – 3.18)	P = 0.14
ED waiting, quartile 3	8 317 (31%)	96 (36%)			1.69 (0.975 – 3.17)	P = 0.075
ED waiting, quartile 4	13 066 48%)	122 (45%)			1.36 (0.788 – 2.54)	P = 0.30
Age quartile 1	7 321 (27%)	44 (16%)			Ref	
Age quartile 2	6 842 (25%)	62 (23%)			1.50 (1.02 – 2.23)	P = 0.039
Age quartile 3	6 642 (24%)	76 (28%)			1.89 (1.30 – 2.76)	P < 0.001
Age quartile 4	6 486 (24%)	87 (32%)			2.19 (1.53 – 3.18)	P < 0.001
COVID-19 hospital occupancy score 1	687 (2.5%)	9 (3.3%)			Ref	
COVID-19 hospital occupancy score 2	2 435 (8.9%)	17 (6.3%)			0.425 (0.188 – 1.02)	P = 0.044
COVID-19 hospital occupancy score 3	8 819 (32%)	89 (33%)			0.513 (0.245 – 1.18)	P = 0.091
COVID-19 hospital occupancy score 4	8 550 (31%)	86 (32%)			0.509 (0.240 – 1.18)	P = 0.094
COVID-19 hospital occupancy score 5	6 800 (25%)	68 (25%)			0.494 (0.230 – 1.16)	P = 0.083

Supplementary Table S9. Binary logistic regression with coagulase-negative staphylococci (CoNS) in 3-4 out of 4 bottles as the outcome. The analysis is based on the dataset CoNS B, 3 785 observations missing data in ED waiting time were excluded, as well as 1 191 observations with CoNS in 1-2 out of 4 bottles. The model includes the variables emergency department waiting time, age and COVID-19 occupancy score. ED = emergency department. 95% CI = 95% confidence interval.

References

1. Statistikdatabas - Socialstyrelsen n.d. https://www.socialstyrelsen.se/statistik-och-data/statistik/statistikdatabasen/. Accessed November 1, 2024.
